# Revisiting life history and morphological proxies for early mammaliaform metabolic rates

**DOI:** 10.1038/s41467-022-32715-0

**Published:** 2022-09-23

**Authors:** Shai Meiri, Eran Levin

**Affiliations:** 1 School of Zoology, Faculty of Life Sciences, Tel Aviv University, Tel Aviv, Israel; 2 The Steinhardt Museum of Natural History, Tel Aviv University, Tel Aviv, Israel

**Keywords:** Palaeontology, Animal physiology

**arising from** E. Newham et al. *Nature Communications* 10.1038/s41467-020-18898-4 (2020)

*Morganucodon* and *Kuehneotherium* are two Late Triassic-Early Jurassic stem mammals that are often portrayed as possessing key mammalian characteristics such as multi-cusped molars^1^, respiratory turbinates, and Harderian glands (associated with grooming and maintaining insulatory pelage^2^). Newham et al.^3^ recently used synchrotron X-ray tomographic imaging of incremental tooth cementum to reconstruct the longevity of large series of *Morganucodon* and *Kuehneotherium*. They conclude that the maximum longevity of these animals was higher than that of similar-sized extant mammals. They infer that these animals must have had low metabolic rates, suggesting ectothermic metabolism, since, they claim, metabolic rates are inversely correlated with longevity. If true, this would mean that mammalian endothermic metabolism evolved tens of millions of years later than currently thought: deep in the Jurassic. We contend that high longevity cannot be taken as a proxy for low metabolic rates. In extant endotherms, the highest metabolic rates characterize birds and bats, two clades that exhibit longer maximum lifespans than terrestrial mammals, which are characterized by lower metabolic rates. Available data likewise suggest that metabolic rates play little role in affecting longevity within and between tetrapod classes once the effects of body size are properly accounted for.

Newham et al.^3^ found that *Morganucodon* lived up to 14 years and *Kuehneotherium* up to 9 years. These values are high compared to similarly-sized extant mammals. Newham et al. thus concluded that they were characterized by low metabolic rates because “In extant tetrapods, negative correlations exist between maximum lifespan and BMR.” While true (if one divides BMR by mass), this statement ignores the predominant role of body size in affecting both BMR and lifespans. In a recent analysis of the longevities of 4100 tetrapod species, including 1,061 mammals and 1,348 reptiles, Stark et al.^4^ found that ectotherms do not live longer than similar size endotherms. Once body mass was accounted, longevity was independent of basal metabolic rates and of resting metabolic rates in tetrapods in general, and in mammals and reptiles alone (for the subset of data with available metabolic rates; n = 662 for BMR). Models with mass alone were superior to models that included both mass and metabolic rates, which were superior to models with metabolic rates alone^4^. For mammals, this was also true when field metabolic rates were used in lieu of BMR. Likewise, mass explains a larger proportion of the variation in mammalian longevity than does BMR^5^. Newham et al. cite only the work of Hulbert et al.^4^ to state that “In extant tetrapods, negative correlations exist between maximum lifespan and BMR”. Hulbert et al.^4^ use mass-specific metabolic rates as a measure for the “rate of living” but in fact suggest that “there are a number of problems associated with presuming a linkage between rate-of-living and maximum life span potential.”

Newham et al.^3^ estimated body masses of 10.7–25.0 g for *Morganucodon* and 14.9–32.7 g for *Kuehneotherium*. In data consisting of 587 amniote species weighing 10-33 g^4^, most species (305, including 36 of 105 mammals) have maximum lifespans of 9 years or longer, and 114 (including 20 mammals) have maximum lifespans of 14 years or longer. Thus, the longevities of *Morganucodon* and *Kuehneotherium* are not particularly high. Stark et al.^4^ found no differences in longevity between endothermic and ectothermic tetrapods when size and phylogeny were accounted for. In non-phylogenetic models, mammals had lower size-corrected longevities than reptiles, but birds, with even faster metabolism^6^, had the highest size-corrected longevities. Newham et al.^3^ recovered a negative relationship between mass-specific metabolic rates and longevity. They also showed that mammals have higher metabolic rates than similar-sized reptiles and higher metabolic rates than reptiles of similar lifespans (their figures 5 C and 6a in ref. 3, respectively). This, however, does not account for the fact that mass-specific metabolic rates, as they acknowledge, decrease with body size. In other words, “size-adjusted metabolic rates” are not independent of size because the relationship is strongly non-isometric (‘Kleiber’s Law’). Hence dividing by size does not remove the effect of mass.

Additional support for the notion of *Morganucodon* as an ectotherm used by Newham et al.^3^ was the ratio between nutrient foramen area and femur length, which is an index for relative blood flow*, Q*_i_. *Q*_i_ was suggested as another proxy for metabolic rate, MMR. Newham et al.^3^ had 69 data points for *Q*_i_, 11 were collected de novo, and 58 were collected by Seymour et al. 2012^7^ (who measured the foramen area directly or from a digital photo of the surface of the bone). They found that *Morganucodon* had a lower *Q*_i_ than expected for a mammal (or for ectothermic varanid reptiles) of a similar size^1^, and considerably closer to small non-varanid reptiles. Seymour et al.^7^ used mammals from ten orders, including bats, in three mammalian infra-classes. *Morganucodon Q*_i_ fits well with a linear regression of *Q*_i,_ on mass in their data. The additional 11 *Q*_i_  values of small mammals, added by Newham et al.^3^, are much higher than expected for mammals of similar sizes from the Seymour et al. dataset^7^. These 11 species form a phylogenetically clustered sample (4 shrews and 7 rodents) and were estimated differently (using a μCT with a 3D imaging software). We suggest that this may have resulted in *Morganucodon* appearing closer to reptiles.

*Morganucodon* and *Kuehneotherium* led long lives for mammals their size. Small mammals at the higher end of the longevity spectrum are mostly bats, which Newham et al.^3^ omitted from their datasets. Small birds are likewise characterized by longer lives than other small tetrapods^4,8^. Flight probably enhances longevity by reducing extrinsic mortality rates (i.e., predation rates). We think this is not a reason to omit flying organisms from the discussion, because extrinsic mortality rates for small mammaliaforms in the Late Triassic and early Jurassic are unknown and could have been substantially lower than modern rates (e.g., no avian, mammalian, or ophidian predators have evolved by then, and carnivorous dinosaurs were probably too large to care). Bats and birds share some of the highest metabolic rates in the animal kingdom, at rest and in the field, and yet live longer than similar-sized terrestrial mammals and reptiles^4,8^. Longevity data are therefore inadequate or at least insufficient for inferring metabolic rates with a reasonable degree of confidence.

Newham et al.^3^ cited evidence for increased metabolic rates in the metabolic scope of mammaliaforms preceding the evolution of *Morganucodon* and *Kuehneotherium* – by nearly 50 million years (i.e., 270-250MA^9–12^), as well as works that are equivocal or suggest later dates^13–16^. Interestingly, one of these^16^ supports the role of turbinates as heat exchange surfaces in *Morganucodo*n, as they do in extant (endothermic) mammals. While far from conclusive, these data are better proxies for metabolic rates than maximum longevities (see also^17^). Based on the admittedly limited paleontological evidence we think it is at least as likely that early mammaliaforms such as *Morganucodon* and *Kuehneotherium* were endotherms rather than ectotherms, and the recent findings regarding their maximum longevities do little to alter this view.
